# Genome characteristics of cluster EA *Microbacterium* bacteriophages HungryHenry, CaptainRex, and ChikPic isolated from soil in Tennessee

**DOI:** 10.17912/micropub.biology.001499

**Published:** 2025-03-19

**Authors:** Sergei Markov, Clayton Barnes, Ariel Hensley, Nicholas More

**Affiliations:** 1 Biology, Austin Peay State University, Clarksville, Tennessee, United States

## Abstract

Bacteriophages HungryHenry, CaptainRex, and ChikPic were isolated from soil collected in Tennessee using the bacterium *Microbacterium foliorum* . These bacteriophages have genomes that are 41,516 bp, 39,941 bp, and 40,333 bp in length and encode 62, 61, and 63 putative genes, respectively. Based on gene content similarity, all three bacteriophages are assigned to actinobacteriophage cluster EA (subclusters EA1, EA2 and EA5, respectively).

**Table 1. Summary of relevant isolation parameters and genome characteristics of EA cluster bacteriophages HungryHenry, CaptainRex, and ChikPic:** All bacteriophages were found around Clarksville, Tennessee

## Description

To expand our understanding of actinobacteriophage diversity and evolution, we described the isolation and characterization of three bacteriophages using *Microbacterium foliorum* ( *M. foliorum* ) NRRL B-24224 (Jacobs-Sera et al., 2020; Hatfull, 2020; Markov et al., 2022; Russell et al., 2019).

Bacteriophages HungryHenry, CaptainRex, and ChikPic were isolated from soil collected in Tennessee using standard methods (Zorawik et al., 2024). Collection site GPS coordinates and ambient temperatures are provided in Table 1. Briefly, soil samples were incubated for 2 hours in PYCa (peptone yeast calcium) liquid medium. The suspensions were then filtered (0.22-µm pores), and the resulting filtrates were inoculated with *M. foliorum* and incubated with shaking (250 rpm) at 30°C for 2 days. An aliquot for each culture was then filtered and plated with PYCa top agar overlays containing *M. foliorum* . Following incubation at 30°C for 48 hours, representative plaques were selected for each sample and bacteriophages were purified through two additional rounds of plating. Bacteriophages for all three samples formed turbid plaques.

DNA was isolated from bacteriophage lysates using the Wizard DNA Clean-Up Kit (Promega, Madison, WI) and enzymatically sheared for sequencing using the Ultra II Library Kit (NEB, Ipswich, MA). An Illumina MiSeq instrument (v3 reagents) was used for DNA sequencing to yield 150-base single-end reads that were assembled using Newbler v2.9 (Russell, 2018) and checked for genomic termini and completeness using Consed v29, as previously described (Russell, 2018). Sequencing results and genome characteristics are provided in Table 1.

Bacteriophage genome annotation was conducted using DNA Master v5.23 (Pope et al., 2018), Glimmer v3.02 (Delcher et al., 1999), GeneMark v2.5p (Besemer and Borodovsky 2005), Aragorn v1.2.41 (Laslett et al., 2004), Phamerator v393.0 (Cresawn et al., 2011), PECAAN v20211202.0 (https://blog.kbrinsgd.org/), BLASTp (Altschul et al., 1990) against the NCBI non-redundant and Actinobacteriophage databases, tRNAscan-SE v2.0 (Lowe, 2016), HHpred v3.2 (Söding et al., 2005) against the PDB_mmCIF70, Pfam v.37.0, and NCBI Conserved Domains databases v3.19. Genes start locations were established using Starterator v485.0 (http://phages.wustl.edu/starterator). Except where otherwise noted, default parameters were used. The number of genes predicted along with putative functional assignments are presented in Table 1. Using the gene content similarity of at least 35% to bacteriophages in the Actinobacteriophage database, HungryHenry, CaptainRex, and ChikPic were all assigned to actinobacteriophage cluster EA, subclusters EA1, EA5, and EA2, respectively (Pope, 2017; Gauthier and Hatfull 2023). When compared to other EA bacteriophages described by Jacobs-Sera et al. (Jacobs-Sera et al., 2020) and in phagesdb.org (https://phagesdb.org/), CaptainRex (jointly with bacteriophage Librie) was found to have the shortest genome of EA5 subcluster. CaptainRex also had the lowest GC content of EA5 bacteriophages, and ChikPic was found to be one of two EA2 bacteriophages without an identifiable tRNA. Transmission electron micrographs for previously characterized cluster EA bacteriophages suggests that all three the bacteriophages are likely to have siphoviral morphologies (Jacobs-Sera et al., 2020).

**Data availability**

GenBank accession numbers for HungryHenry, CaptainRex, and ChikPic are PP978832 , OQ938588, and OR553911 respectively. SRA accession numbers are SRX26311144 , SRX14443488 , and SRX26311141 respectively.
